# Effects of interactions between common genetic variants and smoking on colorectal cancer

**DOI:** 10.1186/s12885-017-3886-0

**Published:** 2017-12-19

**Authors:** Nan Song, Aesun Shin, Hye Soo Jung, Jae Hwan Oh, Jeongseon Kim

**Affiliations:** 1 Cancer Research Institute, Seoul National University College of Medicine, Seoul, South Korea; 2 Department of Preventive Medicine, Seoul National University College of Medicine, 103 Daehak-ro, Jongno-gu, Seoul, 03080 South Korea; 3 Molecular Epidemiology Branch, National Cancer Center, Goyang, South Korea; 4 Center for Colorectal Cancer, National Cancer Center, Goyang, South Korea; 5 Molecular Epidemiology Branch, Division of Cancer Epidemiology and Prevention, Research Institute, National Cancer Center, 323 Ilsan-ro, Insandong-gu, Goyang-si, Gyeonggi-do 10408 South Korea

**Keywords:** Colorectal cancer, Gene-environment interaction, Smoking behaviors, Single-nucleotide polymorphism, Case-control study

## Abstract

**Background:**

Although genome-wide association studies (GWAS) have identified variants in approximately 40 susceptibility loci for colorectal cancer (CRC), there are few studies on the interactions between identified single-nucleotide polymorphisms (SNPs) and lifestyle risk factors. We evaluated whether smoking could modify associations between these genetic variants and CRC risk.

**Methods:**

A total of 703 CRC patients and 1406 healthy controls were included in this case-control study from the National Cancer Center in Korea. Thirty CRC susceptibility SNPs identified in previous GWAS were genotyped. A logistic regression model was used to examine associations between the SNPs and smoking behaviors by sex. The interaction was estimated by including an additional interaction term in the model.

**Results:**

In men, an increased CRC risk was observed for longer durations (OR_>28 vs. ≤28years_ = 1.49 (95% CI = 1.11–1.98)), greater quantities (OR_≥20 vs. <20cigarettes/day_ = 2.12 (1.61–2.79)), and longer pack-years of smoking (OR_≥21 vs. <21pack-years_ = 1.78 (1.35–2.35)). In women, longer pack-years of smoking significantly increased CRC risk (OR_≥5 vs. <5pack-years_ = 6.11 (1.10–34.00)). Moreover, there were significant interactions between smoking status and the polymorphisms rs1957636 at 14q22.3 (*P*_interaction_ = 5.5 × 10^−4^) and rs4813802 at 20p12.3 (*P*_interaction_ = 0.04) in men. Interactions between smoking status and the rs6687758 at 1q41 (*P*_interaction_ = 0.03), duration and the rs174537 at 11q12.2 (*P*_interaction_ = 0.05), and pack-years and the rs4813802 (*P*_interaction_ = 0.04) were also found in women.

**Conclusions:**

Associations between susceptibility SNPs and CRC risk may be modified by smoking behaviors, supporting the existence of gene-smoking interactions.

**Electronic supplementary material:**

The online version of this article (10.1186/s12885-017-3886-0) contains supplementary material, which is available to authorized users.

## Background

Smoking is known to cause many forms of cancer that affect the respiratory, digestive, and urinary tracts [1]. Cigarette smoke contains more than 60 different carcinogenic compounds, including polycyclic aromatic hydrocarbons (PAH), nitrosamines, and aromatic amines, which can form DNA adducts by metabolic activation. This can lead to mutations in tumor-suppressor genes and oncogenes, as well as cell damage, resulting in tumor development [2]. Because carcinogens from smoking can also reach the colorectal mucosa and affect the expression of cancer-related genes [3], it has been established that cigarette smoking is associated with an increased risk of colorectal cancer (CRC) with sufficient evidence by the International Agency of Research on Cancer [4].

Previous studies have reported that cigarette smoke interacts with genetic factors, suggesting that different risk estimates apply to different genetic predispositions [5]. However, there remains a lack of research on gene and smoking interactions for CRC because most studies have focused on genetic polymorphisms of tobacco metabolizing enzymes, and only a weak *mEH3*-smoking interaction effect was found by a meta-analysis [5]. Genome-wide association studies (GWAS) have identified a number of common low-penetrance genetic loci involved in the etiology and progression of CRC [6], but there were few gene-environment interaction studies between GWAS-identified SNPs and smoking [7]. The genome-wide interaction analyses between genetic variants and smoking were also conducted, but none of statistically significant interactions were observed [8, 9].

Although none of GWAS-identified SNPs were directly relevant to tobacco metabolizing enzymes, since the smoking has been the most environmental exposure factors affecting gene-environment interactions in cancer [10] and both of GWAS-identified SNPs and smoking are evident risk factors for CRC, there may be possible indirect gene-environment interactions. In this case-control study, we hypothesized that smoking could modify associations between common genetic variants and CRC risk. To test this hypothesis, we examined the effects of associations between smoking behaviors and 30 susceptibility SNPs, which were previously identified by GWAS, on CRC risk. Interactions between smoking behaviors and the genotypes of the susceptibility SNPs were also investigated.

## Methods

### Study population

Eligible cases included CRC patients who were newly diagnosed and underwent surgical treatment between August 2010 and August 2013 at the National Cancer Center (NCC) in Korea. Among 1427 eligible CRC patients, we were able to contact 1259 patients, and 1070 patients agreed to participate in this study. Among them, 367 patients did not complete our questionnaire and had insufficient blood samples for genotyping; these patients were excluded. Accordingly, a total of 703 CRC patients were included in the analysis. Healthy controls were recruited from a cancer-screening center at the NCC among people who visited for a health check-up program supported by the National Health Insurance Corporation between October 2007 and December 2014. After selecting individuals who completed the questionnaire and had sufficient blood samples, the remaining control subjects were 1:2 frequency-matched to 703 CRC patients according to 5-year interval age and sex. Thus, a total of 703 cases and 1406 controls were included in the analysis. The study was approved by the institutional review board of the NCC (IRB No. NCCNCS-10-350 and NCC 2015–0202).

### Data collection

The CRC patients were face-to-face interviewed by trained interviewers using a structured and written questionnaire (Additional file 1), which was also used in previous studies [11–13]. The original questionnaire written in Korean was developed based on questionnaires of the Korean National Health and Nutrition Examination survey (KNHANES) and the quality assurance and control of the national survey was described in elsewhere [14]. From the questionnaire, we obtained general information on age, sex, family history of CRC, body mass index, education level, and lifestyle information, including alcohol drinking and smoking behavior. The control participants completed self-administered questionnaires on general and lifestyle information. Then, the trained interviewers called them to validate their responses.

Smoking behaviors consisted of ever smoking status, smoking duration, amount of smoking, and pack-years of smoking. The smoking status was classified as never and ever smokers which were defined as those who had smoked ≥5 packs of cigarettes during their lifetime. The pack-years of smoking were calculated by multiplying the amount of smoking (number of cigarettes per day) by duration (number of years smoked) and dividing by 20. The duration, amount, pack-years of smoking was divided into two groups by median value among ever smokers to conduct the gene-environment interaction analyses.

### Genotyping

From the National Human Genome Research Institute (NHGRI) GWAS Catalog [6], we extracted 41 CRC-associated SNPs with *p*-value < 5 × 10^−8^ reported before 2015. Among those SNPs, 14 imputed SNPs were excluded and 9 SNPs were additionally identified through reference review. The 36 susceptibility SNPs were located among 27 loci, which have been identified to be associated with CRC risk by previous GWAS. These SNPs were selected for genotyping (Additional file 2: Table S1) [15–25]. From the subjects’ blood samples, genomic DNA was extracted using a MagAttract DNA Blood M48 kit and BioRobot M48 automatic extraction equipment (Qiagen, Hilden, Germany) according to the manufacturer’s instructions. The genotyping was performed using an Agenabio MassArray iPLEX® gold assay (Agena Bioscience, Inc., San Diego, CA, US). Because of genotyping failure for 4 SNPs and a monomorphic genotype for 2 SNPs, 30 of the originally selected 36 SNPs were included in the final analysis (Additional file 2: Table S2).

### Statistical analysis

The Hardy-Weinberg equilibrium (HWE) was tested for the genotypes of each SNP using a chi-square test for the controls. To compare characteristics between the cases and the controls, a t-test was used for continuous variables, specifically age and body mass index (BMI), and a chi-square test was used for categorical variables, specifically family history of CRC, education level, alcohol drinking, and smoking status. The associations of smoking behaviors and additive SNPs on CRC risk were examined using a logistic regression model that was adjusted for age, family history of CRC, BMI, and education level. The interactions were estimated by including additional interaction (genotypes of each SNP × smoking behaviors) terms in the logistic models. In terms of the statistically significant interactions, we also assessed associations between SNPs and CRC risk after stratification by smoking behavior. For multiple comparisons of the 30 SNPs, false discovery rate (FDR) and Bonferroni tests were additionally conducted. For all association tests, odds ratios (ORs) and 95% confidence intervals (95% CIs) were calculated, and *p*-values less than 0.05 were considered statistically significant. All statistical analyses were stratified by sex and considered two-sided; analyses were performed using SAS version 9.3 (SAS Institute, Inc., Cary, NC, US).

## Results

The characteristics of the study subjects are summarized by sex in Table 1. Because of the frequency matching by age and sex between the cases and the controls, there was no significant difference in age. For the men, those affected by CRC showed a higher frequency of having a family history of CRC (*P* < 0.01), a higher BMI (*P* < 0.01), and a lower education level (*P* < 0.01) than the healthy controls. However, alcohol drinking and smoking statuses were similar among the male CRC patients and the control subjects. For the women, there were no differences in family history of CRC, BMI, or alcohol drinking status, but the CRC patients were more likely to have a lower education level (*P* < 0.01) and more smoking experience than the controls (*P* < 0.01).

**Table 1:** Characteristics of colorectal cancer cases and controls from National Cancer Center in Korea, 2010–2013

Characteristics	Men					Women				
	Case		Control		*P* ^a^	Case		Control		*P* ^a^
	(*N* = 480)		(*N* = 960)			(*N* = 223)		(*N* = 446)		
	N	(%)	N	(%)		N	(%)	N	(%)	
Age (years), mean (SD)	57.0	(9.3)	56.5	(8.9)	0.34	55.1	(9.8)	54.8	(9.4)	0.68
Family history of colorectal cancer^b^					< 0.01					0.21
no	439	(91.5)	919	(95.7)		215	(96.4)	420	(94.2)	
yes	41	(8.5)	41	(4.3)		8	(3.6)	26	(5.8)	
BMI (kg/m^2^), mean (SD)	23.7	(3.0)	24.4	(2.6)	< 0.01	23.8	(4.2)	23.2	(2.8)	0.10
Education level					< 0.01					< 0.01
≤ middle school	151	(31.5)	128	(13.3)		104	(46.6)	69	(15.5)	
≤ high school	194	(40.4)	260	(27.1)		75	(33.6)	197	(44.2)	
≥ college or university	135	(28.1)	562	(58.5)		44	(19.7)	179	(40.1)	
Alcohol drinking					0.19					0.24
never	88	(18.3)	150	(15.6)		124	(55.6)	269	(60.3)	
ever	392	(81.7)	810	(84.4)		99	(44.4)	177	(39.7)	
Smoking					0.06					< 0.01
never	116	(24.2)	191	(19.9)		200	(89.7)	426	(95.5)	
ever	364	(75.8)	769	(80.1)		23	(10.3)	20	(4.5)	

*Abbreviations*: *SD* standard deviation

^a^T-test for continuous variables and chi-square test for categorical variables

^b^First- and/or second-degree relatives with colorectal cancer

Table 2 shows the adjusted associations between smoking behaviors and risk of CRC. The male CRC patients who smoked for more than 28 years (OR = 1.49, 95% CI = 1.11–1.98, *P* < 0.01) at an amount equal or greater than 20 cigarettes per day (OR = 2.12, 95% CI = 1.61–2.79, *P* < 0.01) and who smoked for equal or greater than 21 pack-years (OR = 1.78, 95% CI = 1.35–2.35, *P* < 0.01) were significantly associated with increased risk of CRC. For the women, we found that ever smoking (OR = 2.23, 95% CI = 1.15–4.34, *P* = 0.02) and smoking duration equal or greater than 5 pack-years were associated with increased risk of CRC (OR = 6.11, 95% CI = 1.10–34.00, *P* = 0.04).

**Table 2:** Association between smoking behaviors and risk of colorectal cancer

Smoking behavior	Men							Women						
	Case		Control		OR	(95% CI)^a^	*P*	Case		Control		OR	(95% CI)^a^	*P*
	(*N* = 480)		(*N* = 960)					(*N* = 223)		(*N* = 446)				
	N	(%)	N	(%)				N	(%)	N	(%)			
Smoking status														
never smoker	116	(24.2)	191	(19.9)	1.00	(ref.)		200	(89.7)	426	(95.5)	1.00	(ref.)	
ever (ex- and current) smoker	364	(75.8)	769	(80.1)	0.75	(0.56–1.00)	0.05	23	(10.3)	20	(4.5)	2.23	(1.15–4.34)	0.02
Smoking duration^b^, years (median)														
≤ 28 in men, ≤15 in women	164	(45.1)	422	(54.9)	1.00	(ref.)		8	(34.8)	14	(70.0)	1.00	(ref.)	
> 28 in men, > 15 in women	200	(55.0)	347	(45.1)	1.49	(1.11–1.98)	< 0.01	15	(65.2)	6	(30.0)	4.82	(0.97–24.03)	0.05
Amount of smoking^b^, cigarettes per day (median)														
< 20 in men, < 8 in women	123	(33.8)	419	(54.5)	1.00	(ref.)		9	(39.1)	13	(65.0)	1.00	(ref.)	
≥ 20 in men, ≥ 8 in women	241	(66.2)	350	(45.5)	2.12	(1.61–2.79)	< 0.01	14	(60.9)	7	(35.0)	3.43	(0.73–16.06)	0.12
Pack-years of smoking^b^, pack-years (median)														
< 21 in men, < 5 in women	134	(36.8)	418	(54.4)	1.00	(ref.)		7	(30.4)	12	(60.0)	1.00	(ref.)	
≥ 21 in men, ≥ 5 in women	230	(63.2)	351	(45.6)	1.78	(1.35–2.35)	< 0.01	16	(69.6)	8	(40.0)	6.11	(1.10–34.00)	0.04

*Abbreviations*: *OR* odds ratio, *CI* confidence interval, and *ref.* reference

^a^Logistic regression model adjusted for age, family history of colorectal cancer, BMI, and education level

^b^Smoking behaviors among ever smokers

The associations that were defined between the previously identified common SNPs and the risk of CRC were stratified by sex and provided in Additional file 2: Table S2. We found 5 significant interactions between the common SNPs and the various smoking behaviors assessed for risk of CRC (Table 3). There was an interaction between smoking status and the polymorphism rs1957636 at 14q22.3 in *LOC105370507* for CRC risk in men. The risk allele (C) was associated with decreased risk among never smokers (OR_CC vs. TT_ = 0.36, 95% CI = 0.16–0.79, OR_CT + CC vs. TT_ = 0.54, 95% CI = 0.32–0.92) and increased risk among ever smokers (OR_CC vs. TT_ = 1.51, 95% CI = 1.02–2.24, OR_CT + CC vs. TT_ = 1.33, 95% CI = 1.00–1.77, *P*_interaction for additive model_ = 5.5 × 10^−4^_,_ *P*_interaction for dominant model_ = 1.9 × 10^−3^), with a statistically significant FDR-corrected *p*-value (*P*_interaction for additive model adjusted by FDR_ = 1.8 × 10^−3^). A significant interaction was observed between smoking status and the polymorphism rs4813802 at 20p12.3. This allele was associated with a lower risk of CRC among ever smokers in men (OR_GG vs. TT_ = 0.41, 95% CI = 0.18–0.94, *P*_interaction for additive model_ = 0.04). In women, significant interactions were observed between smoking status and the polymorphism rs6687758 at 1q41 (*P*_interaction for additive model_ = 0.03), smoking duration and the polymorphism rs174537 at 11q12.2 in *MYRF* (*P*_interaction for additive model_ = 0.05), and pack-years of smoking and the polymorphism rs4813802 (*P*_interaction for additive model_ = 0.04), but there were no statistically significant associations between those interactions and risk of CRC.

**Table 3:** Association of GWAS-identified single-nucleotide polymorphisms on risk of colorectal cancer by smoking behaviors

SNP/genotype	Case		Control		OR^a^	(95% CI)	Case		Control		OR^a^	(95% CI)	*P*_interaction_^b^	FDR-corrected *P*_interaction_
	N	(%)	N	(%)			N	(%)	N	(%)				
Men														
Smoking status x rs1957636	never smoker (*N* = 307)						ever smoker (*N* = 1133)							
TT	47	(40.5)	54	(28.3)	1.00	(ref.)	113	(31.0)	285	(37.1)	1.00	(ref.)	5.5 × 10^−4^	1.8 × 10^−3^
CT	54	(46.6)	100	(52.4)	0.62	(0.35–1.08)	178	(48.9)	364	(47.3)	1.27	(0.94–1.72)		
CC	14	(12.1)	37	(19.4)	0.36	(0.16–0.79)	70	(19.2)	118	(15.3)	1.51	(1.02–2.24)		
CT + TT	68	(58.6)	137	(71.7)	0.54	(0.32–0.92)	248	(68.1)	482	(62.7)	1.33	(1.00–1.77)	1.9 × 10^−3^	0.06
Smoking status x rs4813802	never smoker (*N* = 307)						ever smoker (*N* = 1133)							
TT	61	(52.6)	123	(64.4)	1.00	(ref.)	222	(61.0)	463	(60.2)	1.00	(ref.)	0.04	0.55
GT	42	(36.2)	50	(26.2)	1.72	(0.98–3.01)	124	(34.1)	254	(33.0)	1.00	(0.75–1.33)		
GG	7	(6.0)	8	(4.2)	1.27	(0.40–4.06)	8	(2.2)	37	(4.8)	0.41	(0.18–0.94)		
GT + GG	49	(42.2)	58	(30.4)	1.65	(0.97–2.81)	132	(36.3)	291	(37.8)	0.92	(0.70–1.22)	0.05	0.46
Women														
Smoking status x rs6687758	never smoker (*N* = 626)						ever smoker (*N* = 43)							
AA	100	(50.0)	185	(43.4)	1.00	(ref.)	10	(43.5)	8	(40.0)	1.00	(ref.)	0.03	0.34
AG	69	(34.5)	177	(41.6)	0.71	(0.47–1.06)	7	(30.4)	9	(45.0)	1.05	(0.20–5.54)		
GG	9	(4.5)	31	(7.3)	0.49	(0.21–1.15)	5	(21.7)	0	(0.0)	–			
AG + GG	78	(39.0)	208	(48.8)	0.68	(0.46–0.99)					1.86	(0.38–9.20)	0.24	0.53
Smoking duration^c^ x rs174537	≤ 15 years (*N* = 22)						> 15 years (*N* = 21)							
GG	3	(37.5)	4	(28.6)	1.00	(ref.)	7	(46.7)	4	(66.7)	1.00	(ref.)	0.05	0.51
GT	2	(25.0)	7	(50.0)	1.42	(0.05–41.81)	5	(33.3)	2	(33.3)	0.30	(0.01–12.01)		
TT	3	(37.5)	0	(0.0)	–		2	(13.3)	0	(0.0)	–			
GT + TT	5	(62.5)	7	(50.0)	8.55	(0.68–107.61)	7	(46.7)	2	(33.3)	0.36	(0.01–14.35)	0.15	0.99
Pack-years of smoking^c^ x rs4813802	< 5 pack-years (*N* = 19)						≥ 5 pack-years (*N* = 24)							
TT	5	(71.4)	7	(58.3)	1.00	(ref.)	8	(50.0)	6	(75.0)	1.00	(ref.)	0.04	0.57
GT	2	(28.6)	5	(41.7)	0.48	(0.02–13.68)	7	(43.8)	2	(25.0)	20.42	(0.84–499.17)		
GG	0	(0.0)	0	(0.0)	–		1	(6.3)	0	(0.0)	–			
GT + GG	2	(28.6)	5	(41.7)	0.48	(0.02–13.68)	8	(50.0)	2	(25.0)	23.17	(0.96–559.14)	0.03	0.48

*Abbreviations*: *GWAS* genome-wide association study, *SNP* single-nucleotide polymorphism, *OR* odds ratio, *CI* confidence interval, and *FDR* false-discovery rate

^a^Logistic regression model adjusted for age, family history of CRC, BMI, and education level

^b^Logistic regression model including interaction term (smoking behavior × genotypes for SNP)

^c^Smoking behaviors among ever smokers

## Discussion

In this case-control study, we found that various smoking behaviors, including smoking status, smoking duration, amount of smoking, and pack-years of smoking, were associated with risk of CRC. Additionally, we found that associations between several common susceptibility SNPs, including rs1957636 at 14q22.3, rs4813802 at 20p12.3, rs6687758 at 1q41, and rs174537 at 11q12.2, and risk of CRC were modified by smoking behaviors according to sex.

In this study, greater durations, amounts, and pack-years of smoking in men and ever status and greater pack-years of smoking in women were all associated with an increased risk of CRC. A previous meta-analysis also showed an association between smoking and CRC risk in both men and women [26]. Several studies, in contrast, have reported that associations between smoking and CRC risk were attenuated in women due to small sample sizes or the anti-estrogenic effect of smoking [27, 28].

Biological evidence on the association between smoking and CRC has suggested that carcinogenic compounds absorbed from cigarette smoking could cause mutations in the *APC* or *KRAS* genes that are known to be related to early stages of colorectal carcinogenesis [29]. It was reported that *APC* and *KRAS* mutations in colorectal polyps were more frequent among smokers compared to non-smokers [30]. However, there were also inconsistent results on the roles of *APC* and *KRAS* mutations induced by cigarette smoking in CRC [31] as well as a lack of similar studies. Therefore, more studies on the molecular mechanisms that cause genetic damage induced by cigarette smoking in CRC are needed.

Previous studies on gene and smoking interactions in CRC have been based on candidate genes such as *CYP1A1* [32], *CYP1A2* [32], *GPX1* [33], *GSTM1* [32, 34–38], *GSTT1* [35–38], *LEPR* [39], *MAD1L1* [40], *mEH3* [41], *mEH4* [41], *NAT1* [36], *NAT2* [32, 42, 43], *NQO1* [44], *OGG1* [33], *PTEN* [45], *SMAD7* [46], and *TGFBR1* [46]. A meta-analysis reported no evidence for gene and smoking interactions for the *GSTM1*, *GSTT1*, *mEH3*, *mEH4*, and *NAT2* genes in CRC. However, this study suggested a potential negative interaction between smoking and *mEH3* in colorectal adenoma (CRA). There was also a potential positive interaction between smoking and *GSTT1* because smoking was associated with risk of CRA only among *GSTT1*-null carriers [5].

In this study, we identified novel interactions between smoking behaviors and common susceptibility SNPs, specifically rs1957636, rs4813802, rs6687758, rs174537, and rs481302, in CRC according to sex. The most significant interaction was between smoking status and rs1957636 and showed variable effects: allele (C) was associated with decreased or increased risk of CRC according to whether an individual was a never or ever smoker. The SNP rs1957636 is located at 14q22.3 (*LOC105370507*) and is close to the transcription start site of the *BMP4* gene, which is involved in bone morphogenetic protein (BMP) signaling. A similar positive interaction was also observed between rs17563 on *BMP4* and smoking for CRC risk in a previous study [47] in spite of little linkage disequilibrium between rs1957636 and rs17563 (r^2^ = 0.12 in HapMap3 JPT + CHB + CHD individuals). Biologically, BMP signaling has been suggested to cause human cancer through its tumor suppressor properties, but colon cancer cells were resistant to the growth suppression and differentiation induced by BMP4 [48]. Experiments conducted using a rat model showed that BMP4 was up-regulated by chronic cigarette smoking [49]. Thus, it is possible that the interaction between *BMP4* and smoking might explain the variable effects of *BMP4* on the risk of CRC.

For the male subjects, the G allele of the SNP rs4813802 tended to be associated with risk of CRC among the ever smokers, while no associations with the SNP were observed among the subjects who never smoked. A possible interaction between the SNP rs4813802 and smoking on CRC risk was also observed in women. The SNP rs4813802 is located upstream of the *BMP2* gene. Previous experiments have found that higher nicotine concentrations in smokers decreased BMP2 expression [50], which could mediate intestinal cell growth [51]. Furthermore, the *BMP2* gene is part of the transforming growth factor-β (TGFβ) superfamily and plays a role in cell apoptosis, differentiation, and proliferation [52]. However, no results were reported on interactions between SNPs on *BMP2* and smoking behaviors in CRC risk. More studies on BMP pathway loci, including *BMP4* and *BMP2*, should be conducted to explain the missing heritability of CRC [53].

Smoking behaviors also possibly interacted with the polymorphisms rs6687758 at 1q41 (intergenic) and rs174537 at 11q12.2 (*MYRF*) in women, despite the lack of associations with CRC risk. Of these SNPs, rs6687758 is near the *DUSP10* gene, which encodes dual specificity phosphatase 10 (DUSP10). DUSP10 regulates intestinal epithelial cell proliferation through the mitogen-activated protein kinase (MAPK) signaling pathway, thereby acting as a suppressor of CRC [54]. The polymorphism rs174537 is known as an expression quantitative trait locus (eQTL) for the *FADS1* and *FADS2* genes [22], which encode enzymes involved in the metabolism of polyunsaturated fatty acids and mediate the effects of cyclooxygenase-2 (COX-2) in CRC carcinogenesis. Benzo[a]pyrene, one of the carcinogenic compounds included in cigarette smoke, up-regulated COX-2 in mouse cells [55], which in turn could either activate or be dependent on the MAPK pathway, suggesting a possible effect resulting from a gene-smoking interaction [55, 56].

One of the strengths of this study is that we found novel interactions between genes and smoking behaviors that affected CRC risk, accounting for part of the missing heritability in previous GWAS. Especially, the novel interaction between smoking status and the additive genotypes of the polymorphism rs1957636 (*P*_interaction_ = 5.5 × 10^−4^) was still significant after FDR (adjusted *P*_interaction_ = 1.8 × 10^−3^) and Bonferroni adjustments (*P*_interaction_ < 1.67 × 10^−3^). Although several gene-environment interactions involving susceptibility loci identified in GWAS have been evaluated [7, 9, 57–60], no significant gene-smoking interactions have been observed. In addition, this study considered various types of information regarding smoking behavior, such as status, duration, amount, and pack-years of smoking, which differs from most previous gene-smoking interaction studies, which have typically dealt only with smoking status.

A limitation of this study is the insufficient sample size, leading to relatively low statistical power for detecting gene-smoking interactions; a power of 0.66 was found for the additive and dominant models of the SNP rs1957636, with an α = 0.05 in men. To obtain a power over 0.80 for the same condition, a minimum male sample size of 2025 would be recommended. In our analyses of ever smokers, the median values of duration, amount, and pack-years of smoking were defined differently depending on sex. When we analyzed the data using the common median values between the men and the women, the female associations between smoking behaviors and CRC risk were not supportive of further calculations due to the small number of ever smokers. For women, smoking prevalence is very low in Korea [61]. Accordingly, even though we used the female-specific median values for smoking behaviors, several associations between each combination of genotype and smoking behavior and CRC risk could not be calculated.

Another limitation is that this hospital-based case-control study might have had selection bias because the control subjects were recruited from among individuals who took a health examination. However, the control subjects were from the same hospital as the cases, and random sampling and matching with the cases were conducted to reduce the effect of selection bias. Nevertheless, several GWAS-identified SNPs had a higher proportion of risk alleles in controls than in cases. This may be due to ethnic differences in allele frequency of SNPs and potential lack of representativeness of controls who visited hospital for medical-check-up. However, family history of CRC was not that frequent in controls and if controls were actually characterized by higher-risk group for CRC compared to general population, the results would have been estimated towards the null.

Moreover, other potential confounders, such as dietary factors, were not adjusted in the analyses since there were very little difference in the results. Lastly, because we examined SNPs previously identified in GWAS in this analysis, we did not cover or represent all polymorphisms related to CRC risk. GWAS are likely to identify functional genetic variants that are associated with CRC development rather than those correlated with direct disease-causing function. Accordingly, additional fine mapping and functional studies on possible gene-environment interactions should be conducted.

## Conclusions

In conclusion, this study provided evidence that smoking could be associated with CRC risk and identified associations between several common susceptibility SNPs, namely, rs1957636 at 14q22.3, rs4813802 at 20p12.3, rs6687758 at 1q41, and rs174537 at 11q12.2, and CRC risk that may be modified with smoking in CRC carcinogenesis. Further gene-smoking interaction studies with large sample sizes are warranted to confirm our findings.

## Additional files

Additional file 1: Questionnaire we used. (PDF 89 kb)

Additional file 2: Table S1. (Previously identified colorectal cancer susceptibility single-nucleotide polymorphisms by GWAS) and **Table S2.** (**Table S2.** Associations between GWAS-identified single-nucleotide polymorphisms and risk of colorectal cancer). (DOCX 73 kb)

## Abbreviations

95% CIs: 95% confidence intervals
BMI: Body mass index
BMP: Bone morphogenetic protein
COX-2: Cyclooxygenase-2
CRA: Colorectal adenoma
CRC: Colorectal cancer
DUSP10: Dual specificity phosphatase 10
eQTL: Expression quantitative trait locus
FDR: False discovery rate
GWAS: Genome-wide association studies
HWE: Hardy-Weinberg equilibrium
IRB: Institutional review board
MAPK: Mitogen-activated protein kinase
NCC: National Cancer Center
ORs: Odds ratios
PAH: Polycyclic aromatic hydrocarbons
SNPs: Single-nucleotide polymorphisms
TGFβ: Transforming growth factor-β
