# Supplementary material for: Effects of interactions between common genetic variants and smoking on colorectal cancer
Source: BMC Cancer. 2017 Dec 19;17:869. doi: 10.1186/s12885-017-3886-0 (PMC5737484; doi:10.1186/s12885-017-3886-0)
Supplement: Supplementary file 1 — Questionnaire we used. (PDF 89 kb) [file 12885_2017_3886_MOESM1_ESM.pdf]

Health and lifestyle Questionnaire

The number of colorectal cancer patients and accompanying deaths have been increasing in Korea in spite of improvements in early detection and treatment. Several dietary, lifestyle, and genetic factors are considered to influence colorectal cancer risk, while there is a lack of studies that investigate those risk factors in Korean for prevention of the cancer. Therefore, the National Cancer Center is conducting association studies between dietary, lifestyle, and genetic factors and colorectal cancer risk to identify people at high-risk of colorectal cancer and provide evidence-based findings about public environmental interventions for our community to prevent colorectal cancer.

You will be given a separate paper to provide a written consent and this health and lifestyle questionnaire will be handled as such. All data you provide in this questionnaire will be under strict confidentiality. Please read the following questionnaire carefully and fill them out correctly. If you have any questions while you are filling out the questionnaire, please ask the nurses. We appreciate your participation.

(Please answer all questions based the facts <u>before</u> your cancer diagnosis.)

Resistration number		Name	
Sex	Men · Women	Date of Birth	<u>YYYYMMDD</u> (Gregorian · Lunar Calendar)
Date of Visit		Interviewer	

Health Examination

1. If you have ever had any of the examination or operation listed below, please mark the relevant items with a check (√).

Test Yes	Test No	Test item	Reason: Abnormal condition	Reason: Regular examination	No. of tests	Test result: Normal	Test result: Other results	First tested year	Last tested year
□1	□2	Gastroscope	□1	□2		□1	□1 gastritis<br>□2 gastric ulcer<br>□3 esophagitis<br>□4 cancer<br>□5 polyp<br>□6 other ( )		
□1	□2	Upper GI	□1	□2		□1			
□1	□2	Colo-rectal endoscope	□1	□2		□1			
□1	□2	Barium enema	□1	□2		□1			
□1	□2	Fecal occult blood	□1	□2		□1			
□1	□2	Digital rectal exam	□1	□2		□1			
□1	□2	Abdomen supersonic waves	□1	□2		□1			
□1	□2	α-fetoprotein test	□1	□2		□1			

Disease History

2. Have you ever been diagnosed with cancer? (except colorectal cancer)

□1 Yes □2 No (☞ Go to question #3)

(2-1) If yes, please write down which type of cancer and the year in which you were diagnosed. (If there was another type of cancer, or if you were diagnosed with several cancers, please write down them all.)

Type of cancer	Year of diagnosis	Therapy method		
		□1 medication	□2 surgery	□3 immunotherapy
		□4 bone-marrow transplantation	□5 endoscopic exeresis	□6 radiotherapy
		□7 alternative medicine	□8 other ( )	□9 none
		□1 medication	□2 surgery	□3 immunotherapy
		□4 bone-marrow transplantation	□5 endoscopic exeresis	□6 radiotherapy
		□7 alternative medicine	□8 other ( )	□9 none

3. Have you ever been diagnosed of any of the diseases by a doctor?

□1 Yes □2 No (☞ Go to question #4)

(3-1) If yes, please check all that apply and write down the year of diagnosis.

Disease	Full recovery	Undergoing treatment	Ever been treated	Never been treated	First diagnosed year
Colorectal polyp	□1	□2	□3	□4	
Behcet's disease, Crohn's disease, Ulcerative colitis	□1	□2	□3	□4	
Gallstone	□1	□2	□3	□4	
Chelecystitis	□1	□2	□3	□4	

Surgery History

4. Have you ever had any of the surgical operations?

□1 Yes　　□2 No (☞ Go to question #5)

(4-1) If you have ever had any of the operations listed below, please write down the name and year of surgery.

Site	Type of surgery	Year	Site	Type of surgery	Year
Heart			Colon		
Brain			Appendix		
Stomach (include endoscopy)			Hemorrhoids		
Liver			Uterus (except curettage)		
Gallbladder			Ovary		
Kidney, bladder			Breast (include biopsy)		
Pancreas			Other ( )		

Medication

5. Have you ever had to take any of the medications or nutritional supplements listed below regularly in recent 2 year?

□1 Yes (if yes, please write down in detail)　□2 No　□9 Don't know

Type of medication	Total intake amount					Total intake duration
	1~3 pills per week	4~6 pills per week	1 pill per day	2 pills per day	3 pills per day	
Hypotensive drug	□1	□2	□3	□4	□5	___year(s) ___month(s)
Heart medicine	□1	□2	□3	□4	□5	___year(s) ___month(s)
Aspirin	□1	□2	□3	□4	□5	___year(s) ___month(s)
Antiplatelet agent drug	□1	□2	□3	□4	□5	___year(s) ___month(s)
Diabetes medicine	□1	□2	□3	□4	□5	___year(s) ___month(s)
Hyperlipidemia medicine	□1	□2	□3	□4	□5	___year(s) ___month(s)
Anti-ulcer drug	□1	□2	□3	□4	□5	___year(s) ___month(s)

Anti-inflammatory analgesic drug	□1	□2	□3	□4	□5	___year(s) ___month(s)
Nutrition/health supplement	□1	□2	□3	□4	□5	___year(s) ___month(s)
Analgesic (painkiller)	□1	□2	□3	□4	□5	___year(s) ___month(s)
Calcium supplement	□1	□2	□3	□4	□5	___year(s) ___month(s)
Multiple vitamins (Name: ___________)	□1	□2	□3	□4	□5	___year(s) ___month(s)
Single vitamin (Name: ___________)	□1	□2	□3	□4	□5	___year(s) ___month(s)
Others 1 (__________)	□1	□2	□3	□4	□5	___year(s) ___month(s)
Others 2 (__________)	□1	□2	□3	□4	□5	___year(s) ___month(s)

Type	Total intake duration	Type	Total intake duration
Chinese medicine	___year(s) ___month(s)	Folk medicine	___year(s) ___month(s)

(5-1) If you have any supplementary health foods regularly, please mark the relevant items with a check (√).

□1 Iron supplement □2 Chlorella □3 Spirulina □4 Yeast □5 Glucosamine □6 Gamma Linolenic Acid

□7 Lecithin □8 Chitosan □9 Red ginseng □10 Omega-3 □11 DHA/EPA

Alcohol

6. If you ever had a drink containing alcohol, please check the average frequency and amount of alcohol you consumed during the last year.

Type of drink	Never	Average intake frequency for the past year							Average intake amount per once
		1 time per month	2~3 times per month	1 time per week	2~3 times per week	4~6 times per week	1 time per day	2+ times per day	
Beer	□0	□1	□2	□3	□4	□5	□6	□7	_____beer cups(200cc)
Soju	□0	□1	□2	□3	□4	□5	□6	□7	_____Soju glass(50cc)
Western liquid	□0	□1	□2	□3	□4	□5	□6	□7	_____liquid galss(30cc)
Makkoli	□0	□1	□2	□3	□4	□5	□6	□7	_____Makkoli cups(240cc)
Wine	□0	□1	□2	□3	□4	□5	□6	□7	_____wime glass(90cc)
Fruit wine	□0	□1	□2	□3	□4	□5	□6	□7	_____Soju glass(50cc)
Other ( )	□0	□1	□2	□3	□4	□5	□6	□7	_____cup(______cc)

Family History

7. Have any of your family members ever been diagnosed with any type of cancer listed below by a doctor?

☐1 Yes (if yes, please write down in detail) ☐2 No

Type of cancer	Relationship								
	Father	Mother	Brother	Sister	Offspring (son)	Offspring (daughter)	Cousin	Grandfather	Grandmother
	☐1	☐2	☐3	☐4	☐5	☐6	☐7	☐8	☐9
	☐1	☐2	☐3	☐4	☐5	☐6	☐7	☐8	☐9
	☐1	☐2	☐3	☐4	☐5	☐6	☐7	☐8	☐9

8. Have any of your first degree family members ever been diagnosed with any disease listed below by a doctor?

☐1 Yes (if yes, please check all that apply) ☐2 No ☐9 Don't know

DIsease	Relationship					
	Father	Mother	Brother	Sister	Offspring (son)	Offspring (daughter)
Diabetes	☐1	☐2	☐3	☐4	☐5	☐6
Hyperlipidemia	☐1	☐2	☐3	☐4	☐5	☐6
Colorectal polyp	☐1	☐2	☐3	☐4	☐5	☐6
Behcet's disease, Crohn's disease, Ulcerative colitis	☐1	☐2	☐3	☐4	☐5	☐6
Gallstone	☐1	☐2	☐3	☐4	☐5	☐6
Chelecystitis	☐1	☐2	☐3	☐4	☐5	☐6

Physical Activity

Please think about the all vigorous physical activity in the last 7 days. Vigorous physical activity means the activity which makes you lose your breath much more than usual. Please answer the following questions considering activities that you performed at least over 10 minutes per a time.

9. How many days did you perform vigorous activities such as running, aerobic, vigorous bicycling?

__________Day(s) a week ☐0 None (☞ Go to question #10)

(9-1) How long times did you perform vigorous activities in those days?

________hour(s) ________minute(s) a day ☐99 Don'k know

Please think about the all moderate physical activity in the last 7 days. Moderate physical activity means the activity which makes you lose your breath more than usual. Please answer the following questions considering activities that you performed at least over 10 minutes per a time.

10. How many days did you perform moderate physical activities such as lifting light things, tennis, and bicycling? (except walking)

__________Day(s) a week ☐0 None (☞ Go to question #11)

(10-1) How long times did you perform moderate physical activities in those days?

________hour(s) ________minute(s) a day ☐99 Don'k know

Please think about the time of walking in the last 7 days. It includes the walking at the office, house, walking for transportation, exercise, and in the leisure time.

11. How many days did you walk for at least 10 minutes in the last 7 days?

__________Day(s) a week ☐0 None (☞ Go to question #12)

(11-1) How long times did you walk in those days?

________hour(s) ________minute(s) a day ☐99 Don'k know

The followings are questions of your sitting times in the last 7 days. It includes the sitting times at work, house, school, or in the leisure time. It may also include the sitting times such as chatting with friends, reading a book, or watching TV.

12. How long times did you sit in the weekdays last week?

________hour(s) ________minute(s) a day ☐99 Don'k know

13. Do you exercise regularly?

☐1 Yes ☐2 No (☞ Go to question #14)

(13-1) If yes, what kind of exercise do you participate in most frequently?

	Type of exercise	Average exercise time (per week)
1		_____hour(s) _____minute(s) ☐99 Dont' know
2		_____hour(s) _____minute(s) ☐99 Dont' know
3		_____hour(s) _____minute(s) ☐99 Dont' know

Dietary Habits

14. Do you have your meal more than 10 minutes, on average?

☐1 Yes ☐2 No

15. Do you eat vegetables (except kimchi), seaweeds, or mushrooms for every meal?

☐1 Yes ☐2 No

16. Do you eat burnt meat?

☐1 Never ☐2 Sometimes ☐3 Often ☐4 Don't eat meat

General Information

17. Now, we will ask about your height and weight.

(17-1) What are your height and weight in current? _______kg _______cm

(17-2) What was your weight two years ago? _______kg ☐99 Don't know

(17-3) What was your weight at your age 35? _______kg ☐99 Don't know

(17-4) What was your weight at your age 18? _______kg ☐99 Don't know

※ Questions for women only

18. Please write down your age at menarche and age at start of regular menstruation cycle.

- Age at menarche _______years old ☐0 Not started yet
- Age at start of regular menstruation cycle _______years old ☐0 Not Don'k know

(18-1) Is your menstruation cycle regular?

☐1 Yes (_____days on average, ex: 28 days) ☐2 No

(18-2) Is your duration of each menstruation period regular?

☐1 Yes (_____days on average, ex: 7 days) ☐2 No

(18-3) When did you have your last menstruation?

_____(month) _____(day) ☐1 Menopausal

19. Please answer the following questions for postmenopausal women only.

(19-1) Please write down your age at menopause and the reason.

- Age at menopause _______years old
- Reason

☐1 Natural menopause ☐2 Surgery (hysterectomy)

☐3 Radiotherapy ☐4 Medication ☐5 Other _________

(19-2) Have you used hormone replacement therapy after menopause?

☐1 Yes (current use) Duration of use: ____year(s) ____month(s)

☐2 Yes (used before, not current) Duration of use: ____year(s) ____month(s)

☐3 Never

20. Please write down about your fertility history if relevant.

- Total number of pregnancy ________
- Number of natural abortion ________
- Number of artificial abortion ________
- Number of stillbirth ________
- Number of normal delivery ________
- Number of cesarean ________

(20-1) Please write down your ages at the first pregnancy, full term normal delivery (week 37~42), and the last delivery.

- First pregnancy _______years old
- First full term normal delivery _______years old
- Last delivery _______years old

(20-2) What was the result of your first pregnancy?

□1 Normal delivery □2 Stillbirth (deadborn) □3 Abortion by extrauterine pregnancy □4 Natural abortion □5 Artificial abortion

(20-3) Have you ever been diagnosed with gestational diabetes?

□1 Yes □2 Never □9 Don't know

(20-4) Have you ever been diagnosed with gestational hypertension?

□1 Yes □2 Never □9 Don't know

21. Have you ever breast-fed? □1 Yes □2 No □9 Don't know

Please write down if relevant.

(21-1) Number of child you breast-fed in Total _______ □99 Don't know

(21-2) Your age at first breast-fed _______years old □99 Don't know

(21-3) Duration of the first breast-fed _______month(s) □99 Don't know

(21-4) Duration of total breast-fed Total _______month(s) □99 Dont' know

22. Have you ever used oral contraceptives?

□1 Never □2 Yes, but not current □3 Yes, I still use it. □9 Don't know

(23-1) At what age you start to use oral contraceptives?

_______years old □99 Don't know

(23-2) How long did you use oral contraceptives?

_______year(s) _______month(s) □99 Don't know

(Additional questionnaire)

Disease History

1. Have you ever been diagnosed of any of the diseases by a doctor? (except cancer)

☐1 Yes ☐2 No (☞ Go to question #2)

(1-1) If yes, please check all that apply and write down the year of diagnosis.

Disease	Full recovery	Undergoing treatment	Ever been treated	Never been treated	First diagnosed year
Hypertension	☐1	☐2	☐3	☐4	
Diabetes	☐1	☐2	☐3	☐4	
Hyperlipidemia	☐1	☐2	☐3	☐4	
Myocardial Infarction	☐1	☐2	☐3	☐4	
Angina	☐1	☐2	☐3	☐4	
Arrhythmia	☐1	☐2	☐3	☐4	
Stroke	☐1	☐2	☐3	☐4	
Gastritis	☐1	☐2	☐3	☐4	
Gastric ulcer	☐1	☐2	☐3	☐4	
Duodenal ulcer	☐1	☐2	☐3	☐4	
Irritable bowel syndrome	☐1	☐2	☐3	☐4	
Hepatosis	☐1	☐2	☐3	☐4	
Fatty liver	☐1	☐2	☐3	☐4	
Hepatitis type B	☐1	☐2	☐3	☐4	
Hepatitis type C	☐1	☐2	☐3	☐4	
Hepatocirrhosis	☐1	☐2	☐3	☐4	
Other ( )	☐1	☐2	☐3	☐4	

Smoking and Alcohol

2. Have you smoked at least 5 packs of cigarettes (100 cigarettes) in your entire life?

☐1 No (nonsmoker) (☞ Go to question #4)

☐2 Yes, but I quit (ex-smoker). (☞ Go to question #2-1)

☐3 Yes, and I still smoke (current smoker) (☞ Go to question #2-2)

(2-1) (Questions for ex-smoker only) How long has it been since you quit smoking cigarettes?

☐1 Less than 6 months

☐2 6-12 months

☐3 More than 1 year : ( )year(s) (☞ Go to question #3)

(2-2) (Questions for current smoker only) Do you have plan to quit smoking?

☐1 Yes, in the next 1 month.

☐2 Yes, in the next 6 months.

☐3 Yes, more than 6 months

☐4 No (☞ Go to question #3)

3.(Questions for both ex-smoker and current smoker) Please answer the questions below.

- At what age did you start smoking? Age _______
- How many years have you been smoking? Total _______year(s)
- On average, how many cigarettes or packs do you smoke per day?

About _______cigarette(s) or ________pack(s)

4. Do you currently drink alcohol beverages?

☐1 Never

☐2 Yes, former (☞ Go to question #4-1)

☐3 Yes, current

(4-1) (Questions for former drinker only) How long has it been since you quit drinking alcoholic beverages?

☐1 Less than 6 months

☐2 6-12 months

☐3 More than 1 year : (   )year(s)

General Information

5.Which statement corresponds to your current marital status?

☐1 Not married ☐2 Married and/or Living together ☐3 Separated

☐4 Widowed ☐5 Divorced ☐6 Other

6.What is your educational attainment?

☐1 Did not go to school or did not graduate from elementary school

☐2 Graduated from elementary school

☐3 Graduated from middle school

☐4 Graduated from high school

☐5 More than college (university)

7. Which statement describes your current employment situation?

☐1 Professional (Legislators, cleric, artist, health care provider, etc.)

☐2 Administrator (Senior officials, corporate managers, general managers, etc.)

☐3 Office worker (General office worker, customer-service office worker, etc.)

☐4 Sales

☐5 Service worker

☐6 Agricultural worker, Forestry and related worker, Fishery worker

☐7 Manual worker

☐8 Soldier ☐9 Miner ☐10 Unemployed ☐11 House maker ☐12 Other

8. What is your estimate of your average monthly household income? (Korean won)

☐$_1$ Less than 1,000,000

☐$_2$ 1,000,000~<2,000,000

☐$_3$ 2,000,000~<3,000,000

☐$_4$ 3,000,000~<4,000,000

☐$_5$ Equal or more than 4,000,000
