# Supplementary material for: Effects of interactions between common genetic variants and smoking on colorectal cancer
Source: BMC Cancer. 2017 Dec 19;17:869. doi: 10.1186/s12885-017-3886-0 (PMC5737484; doi:10.1186/s12885-017-3886-0)
Supplement: Supplementary file 2 — (Previously identified colorectal cancer susceptibility single-nucleotide polymorphisms by GWAS) and Table S2. (Table S2. Associations between GWAS-identified single-nucleotide polymorphisms and risk of colorectal cancer). (DOCX 73 kb) [file 12885_2017_3886_MOESM2_ESM.docx]

| Table S1. Previously identified colorectal cancer susceptibility single-nucleotide polymorphisms by GWAS | | | | | | | |
| --- | --- | --- | --- | --- | --- | --- | --- |
| SNP | Cytogenetic  region | Chromosomal  Location | Mapped gene | Reported gene | Allele^a^ | | Ref. |
|  |  |  |  |  | A1 | A2 |  |
| rs6691170 | 1q41 | 221872104 | *intergenic* | *DUSP10* | T | G | Houlston et al. Nat Genet. 2010 |
| rs6687758 | 1q41 | 221991606 | *intergenic* | *DUSP10, intergenic* | G | A | Houlston et al. Nat Genet. 2010 |
| rs10936599 | 3q26.2 | 169774313 | *MYNN* | *MYNN, TERC, ACTRT3, LRRC34, intergenic* | T | C | Houlston et al. Nat Genet. 2010 |
| rs647161 | 5q31.1 | 135163402 | *C5orf66* | *PITX1* | A | C | Jia et al. Nat Genet. 2012 |
| rs7758229 | 6q25.3 | 160419220 | *SLC22A3* | *SLC22A3* | T | G | Cui et al. Gut. 2011 |
| rs16892766 | 8q23.3 | 116618444 | *intergenic* | *EIF3H* | C | A | Tomlinson et al. Nat Genet. 2008 |
| rs6983267 | 8q24.21 | 127401060 | *CASC8, CCAT2* | *intergenic* | T | G | Tomlinson et al. Nat Genet. 2007 |
| rs7014346 | 8q24.21 | 127412547 | *CASC8* | *POU5FIP1, HsG57825, DQ515897* | G | A | Tenesa et al. Nat Genet. 2008 |
| rs10505477 | 8q24.21 | 127395198 | *CASC8* | *ORF, DQ515897, MYC* | G | A | Zanke et al. Nat Genet. 2007 |
| rs719725 | 9p24 | 6365683 | *intergenic* | - | C | A | Zanke et al. Nat Genet. 2007 |
| rs10795668 | 10p14 | 8659256 | *LOC105376400* | *intergenic* | A | G | Tomlinson et al. Nat Genet. 2008 |
| rs704017 | 10q22.3 | 79059375 | *ZMIZ1-AS1* | *AS1, ZMIZ1* | G | A | Zhang et al. Nat Genet. 2014 |
| rs11196172 | 10q25.2 | 112967084 | *TCF7L2* | *TCF7L2* | A | G | Zhang et al. Nat Genet. 2014 |
| rs1665650 | 10q25.3 | 116727589 | *HSPA12A* | *HSPA12A* | C | T | Jia et al. Nat Genet. 2012 |
| rs174537 | 11q12.2 | 61785208 | *MYRF* | *MYRF, FADS1, FADS2, FEN1* | T | G | Zhang et al. Nat Genet. 2014 |
| rs4246215 | 11q12.2 | 61796827 | *FEN1* | *FEN1, C11orf9, FADS1, FADS2* | T | G | Zhang et al. Nat Genet. 2014 |
| rs174550 | 11q12.2 | 61804006 | *FADS1* | *FADS1* | T | C | Zhang et al. Nat Genet. 2014 |
| rs1535 | 11q12.2 | 61830500 | *FADS2* | *FADS2, FEN FADS1* | A | G | Zhang et al. Nat Genet. 2014 |
| rs3802842 | 11q23.1 | 111300984 | *COLCA1, COLCA2* | *LOC120376, intergenic* | A | C | Tenesa et al. Nat Genet. 2008 |
| rs10849432 | 12p13.31 | 6276561 | *intergenic* | *PLEKHG6, TNFRSF1A, CD9* | T | C | Zhang et al. Nat Genet. 2014 |
| rs10774214 | 12p13.32 | 4259186 | *CCND2-AS1* | *CCND2, C12orf5, FGF6, RAD51AP1, FGF23, PARP11* | C | T | Jia et al. Nat Genet. 2012 |
| rs11169552 | 12q13.12 | 50761880 | *ATF1, LOC105369765* | *DIP2B, ATF1* | T | C | Houlston et al. Nat Genet. 2010 |
| rs7136702 | 12q13.13 | 50486433 | *intergenic* | *intergenic* | C | T | Houlston et al. Nat Genet. 2010 |
| rs4444235 | 14q22.2 | 53944201 | *intergenic* | *BMP4* | C | T | Houlston et al. Nat Genet. 2008 |
| rs1957636 | 14q22.3 | 54093300 | *LOC105370507* | *-* | C | T | Tomlinson et al. Nat Genet. 2008 |
| rs4779584 | 15q13.3 | 32702555 | *intergenic* | *intergenic, GREM1, SCG5, CRAC1, HMPS* | C | T | Tomlinson et al. Nat Genet. 2008 |
| rs9929218 | 16q22.1 | 68787043 | *CDH1* | *CDH1* | A | G | Houlston et al. Nat Genet. 2008 |
| rs12603526 | 17p13.3 | 897353 | *intergenic* | *NXN* | C | T | Zhang et al. Nat Genet. 2014 |
| rs7229639 | 18q21.1 | 48924606 | *SMAD7* | *SMAD7* | G | A | Zhang et al. Nat Genet. 2014 |
| rs10411210 | 19q13.11 | 33041394 | *RHPN2* | *RHPN2* | T | C | Houlston et al. Nat Genet. 2008 |
| rs1800469 | 19q13.2 | 41354391 | *B9D2, TGFB1* | *TGFB1, B9D2* | G | A | Zhang et al. Nat Genet. 2014 |
| rs2241714 | 19q13.2 | 41363487 | *B9D2, TMEM91* | *TGFB1, B9D2* | C | T | Zhang et al. Nat Genet. 2014 |
| rs961253 | 20p12.3 | 6423634 | *intergenic* | *intergenic* | A | C | Houlston et al. Nat Genet. 2008 |
| rs4813802 | 20p12.3 | 6718948 | *intergenic* | *BMP2* | G | T | Peters et al. Gastroenterology. 2012 |
| rs2423279 | 20p12.3 | 7831703 | *intergenic* | *HAO1, PLCB1* | C | T | Jia et al. Nat Genet. 2012 |
| rs4925386 | 20q13.33 | 62345988 | *LAMA5* | *LAMA5, intergenic* | C | T | Houlston et al. Nat Genet. 2010 |
| Abbrevations: GWAS (genome-wide association study), SNP (single-nucleotide polymorphism), Ref. (reference), NCBI (National Center for Biotechnology Information), and dbSNP (Database of Single Nucleotide Polymorphisms). | | | | | | | |
| ^a^A1 is risk and A2 is reference allele according to NCBI dbSNP. | | | | | | | |

| Table S2. Associations between GWAS-identified single-nucleotide polymorphisms and risk of colorectal cancer | | | | | | | | | | | |
| --- | --- | --- | --- | --- | --- | --- | --- | --- | --- | --- | --- |
| SNP | Cytogenetic  region | Mapped gene | Allele^a^ | | Men | | |  | Women | | |
|  |  |  | A1 | A2 | OR^b^ | (95% CI) | *P* |  | OR^b^ | (95% CI) | *P* |
| rs6687758 | 1q41 | *intergenic* | G | A | 1.35 | (1.11-1.65) | 2.7×10^-3^ |  | 0.80 | (0.60-1.08) | 0.15 |
| rs10936599 | 3q26.2 | *MYNN* | T | C | 0.95 | (0.80-1.13) | 0.57 |  | 1.17 | (0.91-1.50) | 0.23 |
| rs647161 | 5q31.1 | *C5orf66* | A | C | 1.25 | (1.04-1.50) | 0.02 |  | 1.29 | (0.98-1.69) | 0.07 |
| rs7758229 | 6q25.3 | *SLC22A3* | T | G | 1.05 | (0.85-1.29) | 0.62 |  | 1.17 | (0.87-1.58) | 0.30 |
| rs6983267 | 8q24.21 | *CASC8, CCAT2* | T | G | 0.82 | (0.69-0.97) | 0.02 |  | 0.76 | (0.59-0.97) | 0.03 |
| rs7014346 | 8q24.21 | *CASC8* | G | A | 0.84 | (0.70-1.01) | 0.06 |  | 0.75 | (0.57-0.98) | 0.03 |
| rs10505477 | 8q24.21 | *CASC8* | G | A | 0.82 | (0.70-0.97) | 0.02 |  | 0.72 | (0.56-0.93) | 9.8×10^-3^ |
| rs10795668 | 10p14 | *LOC105376400* | A | G | 0.79 | (0.67-0.94) | 0.01 |  | 0.92 | (0.70-1.19) | 0.51 |
| rs704017 | 10q22.3 | *ZMIZ1-AS1* | G | A | 1.23 | (1.03-1.46) | 0.02 |  | 1.16 | (0.89-1.50) | 0.27 |
| rs11196172 | 10q25.2 | *TCF7L2* | A | G | 1.19 | (0.98-1.44) | 0.09 |  | 1.14 | (0.86-1.51) | 0.37 |
| rs1665650 | 10q25.3 | *HSPA12A* | C | T | 1.13 | (0.94-1.36) | 0.18 |  | 0.97 | (0.74-1.28) | 0.83 |
| rs174537 | 11q12.2 | *MYRF* | T | G | 0.80 | (0.66-0.95) | 0.01 |  | 0.88 | (0.67-1.17) | 0.38 |
| rs174550 | 11q12.2 | *FADS1* | T | C | 1.21 | (1.01-1.45) | 0.04 |  | 1.11 | (0.84-1.46) | 0.48 |
| rs1535 | 11q12.2 | *FADS2* | A | G | 1.21 | (1.01-1.45) | 0.04 |  | 1.13 | (0.85-1.49) | 0.40 |
| rs3802842 | 11q23.1 | *COLCA1, COLCA2* | A | C | 1.06 | (0.89-1.25) | 0.52 |  | 0.89 | (0.69-1.15) | 0.37 |
| rs10849432 | 12p13.31 | *intergenic* | T | C | 1.08 | (0.87-1.35) | 0.47 |  | 1.16 | (0.83-1.62) | 0.38 |
| rs10774214 | 12p13.32 | *CCND2-AS1* | C | T | 0.91 | (0.77-1.08) | 0.28 |  | 0.90 | (0.70-1.16) | 0.41 |
| rs11169552 | 12q13.12 | *ATF1, LOC105369765* | T | C | 1.12 | (0.94-1.33) | 0.22 |  | 0.83 | (0.64-1.09) | 0.17 |
| rs7136702 | 12q13.13 | *intergenic* | C | T | 1.04 | (0.88-1.24) | 0.64 |  | 0.90 | (0.70-1.15) | 0.41 |
| rs4444235 | 14q22.2 | *intergenic* | C | T | 1.04 | (0.88-1.22) | 0.65 |  | 0.90 | (0.71-1.15) | 0.41 |
| rs1957636 | 14q22.3 | *LOC105370507* | C | T | 1.06 | (0.89-1.25) | 0.53 |  | 1.27 | (0.99-1.63) | 0.06 |
| rs4779584 | 15q13.3 | *intergenic* | C | T | 0.88 | (0.69-1.12) | 0.29 |  | 0.69 | (0.48-0.98) | 0.04 |
| rs9929218 | 16q22.1 | *CDH1* | A | G | 1.14 | (0.91-1.44) | 0.26 |  | 1.31 | (0.93-1.83) | 0.12 |
| rs12603526 | 17p13.3 | *intergenic* | C | T | 0.94 | (0.79-1.13) | 0.52 |  | 1.00 | (0.77-1.29) | 0.99 |
| rs10411210 | 19q13.11 | *RHPN2* | T | C | 0.77 | (0.62-0.96) | 0.02 |  | 0.68 | (0.48-0.95) | 0.02 |
| rs1800469 | 19q13.2 | *B9D2, TGFB1* | G | A | 1.11 | (0.94-1.31) | 0.22 |  | 0.86 | (0.67-1.11) | 0.26 |
| rs2241714 | 19q13.2 | *B9D2, TMEM91* | C | T | 1.09 | (0.93-1.29) | 0.28 |  | 0.90 | (0.70-1.16) | 0.42 |
| rs961253 | 20p12.3 | *intergenic* | A | C | 1.41 | (1.06-1.86) | 0.02 |  | 1.17 | (0.78-1.77) | 0.45 |
| rs4813802 | 20p12.3 | *intergenic* | G | T | 0.98 | (0.79-1.20) | 0.82 |  | 1.18 | (0.87-1.60) | 0.28 |
| rs2423279 | 20p12.3 | *intergenic* | C | T | 1.19 | (0.99-1.43) | 0.07 |  | 1.17 | (0.90-1.52) | 0.25 |
| Abbreviations: GWAS (genome-wide association study), SNP (single-nucleotide polymorphism), RAF (risk allele frequency), HWE (Hardy-Weinberg Equilibrium), OR (odds ratio), CI (confidence interval). | | | | | | | | | | | |
| ^a^A1 is risk and A2 is reference allele according to NCBI dbSNP. | | | | | | | | | | | |
| ^b^Additive effect by multivariate logistic regression model adjusted for age, sex, family history of CRC, BMI, and education level. | | | | | | | | | | | |
